# EPODE approach for childhood obesity prevention: methods, progress and international development

**DOI:** 10.1111/j.1467-789X.2011.00950.x

**Published:** 2012-04

**Authors:** J-M Borys, Y Le Bodo, S A Jebb, J C Seidell, C Summerbell, D Richard, S De Henauw, L A Moreno, M Romon, T L S Visscher, S Raffin, B Swinburn, the EEN Study Group

**Affiliations:** 1 EPODE European Network Coordinating TeamProteines, Paris, France; 2 MRC Human Nutrition Research, Elsie Widdowson LaboratoryCambridge, UK; 3 Institute of Health Sciences, Faculty of Earth and Life Sciences, VU UniversityAmsterdam, the Netherlands; 4 School of Medicine and Health, Durham UniversityStockton-on-Tees, UK; 5 Quebec Heart and Lung InstituteQuebec, Canada; 6 Ghent University – Department of Public Health, University HospitalGent, Belgium; 7 GENUD ‘Growth, Exercise, Nutrition and Development’ Research Group, School of Health Sciences (EUCS), University of ZaragozaZaragoza, Spain; 8 Department of Nutrition, Faculty of MedicineLille, France; 9 Research Centre for the Prevention of Overweight Zwolle, VU-WindesheimZwolle, the Netherlands; 10 Link-UpParis, France; 11 WHO Collaborating Centre for Obesity Prevention, Deakin UniversityMelbourne, Australia

**Keywords:** Children, EPODE, obesity, prevention

## Abstract

Childhood obesity is a complex issue and needs multistakeholder involvement at all levels to foster healthier lifestyles in a sustainable way. ‘**E**nsemble **P**révenons l'**O**bésité**D**es **E**nfants’ (EPODE, *Together Let's Prevent Childhood Obesity*) is a large-scale, coordinated, capacity-building approach for communities to implement effective and sustainable strategies to prevent childhood obesity. This paper describes EPODE methodology and its objective of preventing childhood obesity.

At a central level, a coordination team, using social marketing and organizational techniques, trains and coaches a local project manager nominated in each EPODE community by the local authorities. The local project manager is also provided with tools to mobilize local stakeholders through a local steering committee and local networks. The added value of the methodology is to mobilize stakeholders at all levels across the public and the private sectors. Its critical components include political commitment, sustainable resources, support services and a strong scientific input – drawing on the evidence-base – together with evaluation of the programme.

Since 2004, EPODE methodology has been implemented in more than 500 communities in six countries. Community-based interventions are integral to childhood obesity prevention. EPODE provides a valuable model to address this challenge.

## Introduction

The prevalence of overweight and obesity has increased worldwide during the last 30 years (1–6). Although there are signs of stabilization in children in some age groups in certain countries (7,8), large-scale, effective prevention of overweight and obesity remains a pressing public health priority given the adverse impact on health and quality of life in childhood (9–12) and the increased risk of obesity and associated health complications in adulthood. Nearly two-thirds of children with obesity will continue to suffer from the condition throughout their adult life (13).

There is a clear need to bridge the gap between awareness of the problem and practical implementation of necessary lifestyle changes. Over the past 10 years, several studies have provided evidence that the prevention of obesity in children is possible through interventions aimed at modifying eating habits and increasing physical activity (14–16).

Of particular note is a long-term school-based nutrition education programme – the Fleurbaix Laventie Ville Santé (FLVS) study. This study was initiated in 1992 in two towns in the north of France – Fleurbaix and Laventie. (In 1991 the two towns totalled 6,500 inhabitants.) This was followed by a number of intervention programmes over the following 12 years (17). A comparison population was selected from two other towns, also situated in northern France and of similar demographic and socioeconomic characteristics. The FLVS study showed a substantial decrease in the prevalence of overweight in children (1992: 11.4% in FLVS and 12.6% in the two comparison towns, *P* = 0.6; 2004: 8.8% in FLVS and 17.8% in the two comparison towns, *P* ≤ 0.0001) although it was 8 years before the decline in prevalence became apparent. A key learning from this study was that the intervention programme was effective across all socioeconomic levels (17). However, it was apparent that interventions targeting schools alone were not sufficient, and that progress was only made when the mobilization of the population became more generalized at community level and involved schools, pre-schools, local sports and parents associations, catering structures, health professionals, elected representatives, and local stakeholders from the public and private sectors (17). The process evaluation of the intervention in FLVS identified four critical pillars for a large-scale community-based intervention (CBI). These ‘4 pillars’ formed the basis of Ensemble Prévenons l'Obésité Des Enfants (EPODE) methodology (further discussed in objectives).

EPODE was first launched in 2004 in ten French pilot communities, in line with official French guidelines on diet and physical activity (18). EPODE has since expanded to more than 500 communities worldwide. EPODE methodology promotes the involvement of multiple stakeholders at two levels: (i) at a central level (ministries, health groups, non-governmental organizations [NGOs] and private partners); and (ii) at a local level (political leaders, health professionals, families, teachers, local NGOs and the local business community) (19).

Monitoring and evaluation are crucial for the success of the programme. EPODE practices include outcome measurements, together with process and output indicators at central, local and individual levels.

The EPODE European Network (EEN) involves four European universities: the Free University of Amsterdam (the Netherlands), the University of Ghent (Belgium), the University of Lille 2 (France), and the University of Saragossa (Spain), as well as an International Scientific Advisory Board. Since 2008, the EEN has been working to enrich EPODE methodology and disseminate practical experience in the implementation of similar initiatives in other countries (20).

The aim of this paper is to provide a detailed description of EPODE methodology, including its broad and overarching approach to strengthening and enriching CBIs aimed at preventing childhood obesity.

## Ensemble Prévenons l'Obésité Des Enfants structure and methodology

### Definition

EPODE is a coordinated, capacity-building approach aimed at reducing childhood obesity through a societal process in which local environments, childhood settings and family norms are directed and encouraged to facilitate the adoption of healthy lifestyles in children (i.e. the enjoyment of healthy eating, active play and recreation).

EPODE philosophy is based on multiple components, including a positive approach to tackling obesity, with no cultural or societal stigmatization; step-by-step learning, and an experience of healthy lifestyle habits, tailored to the needs of all socioeconomic groups.

Primary EPODE target groups are children aged 0–12 years and their families. Local stakeholders are also targeted. Through initiatives and a long-term programme, stakeholders foster and promote healthy lifestyles in families in a sustainable manner.

### Objectives

The FLVS study identified four critical factors which now form the four pillars of the EPODE methodology:

1. Political commitment: Gaining formal political commitment at central and local levels from the leaders of the key organization(s), which influence national, federal or state policies as well as local policies, environments and childhood settings;
2. Resources: Securing sufficient resources to fund central support services and evaluation, as well as contributions from local organizations to fund local implementation;
3. Support services: Planning, coordinating, and providing the social marketing, communication and support services for community practitioners and leaders;
4. Evidence: Using evidence from a wide variety of sources to inform the delivery of EPODE and to evaluate process, impact and outcomes of the EPODE programme.

Building on the lessons learned from the FLVS study and other successful CBIs, EPODE methods have evolved from 2004 as a result of a heuristic process and continuous improvement dynamics. The methods described here have been shaped over 6 years of implementation in France. Throughout the paper, the use of the term ‘central level’ refers to a country although in some instances, this may be a state or a region; ‘local level’ refers to a community, which can mean a town, part of a larger city, a village or collection of villages.

### Stakeholders

**Figure 1:**
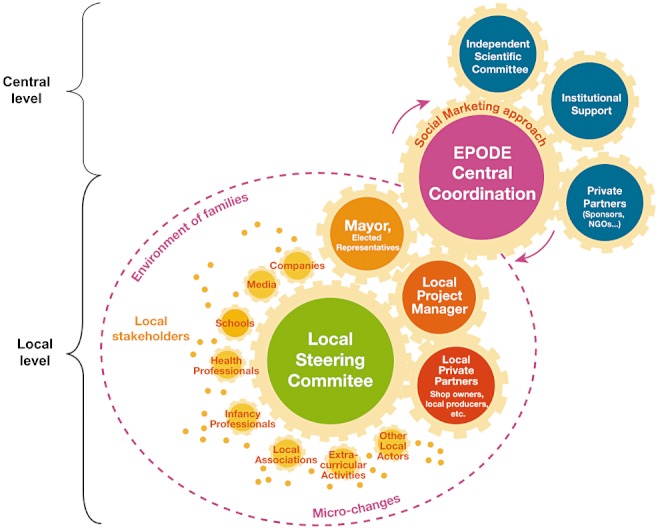
Ensemble Prévenons l'Obésité Des Enfants (EPODE) stakeholders at central and local levels. NGO, non-governmental organization.

In each country, EPODE fosters both a ‘top-down’ leadership and a ‘bottom-up’ mobilization of support (see Fig. 1). EPODE methodology promotes the involvement of multiple stakeholders at central level (e.g. with endorsement from ministries, and support from health groups, NGOs and private partners) and the programme benefits from the expertise and guidance of an independent expert committee (a group of specialists and academics from different fields, playing an advisory role to the central coordination of the programme).

To put EPODE methodology into practice, a Central Coordination Team (CCT), using social marketing and organizational techniques, trains and coaches local project managers appointed in each community by the mayor (or other local leader able to champion the programme). The role of the local project manager is to mobilize a wide diversity of local stakeholders (see Supporting Information Appendix S1), especially in schools, pre-schools, extra-curricular organizations and any social network of associations, which are key settings to implement activities with children and families. For this purpose, the local project manager coordinates a multifaceted local steering committee of local representatives from various fields (elected representatives, heads of municipal services, school professionals and local partners). This committee meets on a regular basis to make key decisions, foster the implementation of activities and actions, and generate peer-to-peer dynamics.

### Principles

EPODE pillars have been subdivided into ten EPODE implementation principles, which describe the EPODE methodology:

1. Each country (or region) commits to a central coordination support/capacity;
2. Each local community has a formal political commitment for several years from the outset;
3. Each local community has a dedicated local project manager with sufficient capacity and cross-sectoral mandate for action;
4. A multistakeholder approach is integral to the central and local structures and processes;
5. An approach to action is planned and coordinated using social marketing. This is specifically to define a series of themed messages and actions, informed by evidence, from a wide variety of sources, and in line with official recommendations;
6. Local stakeholders are involved in the planning processes and are trusted with sufficient flexibility to adapt actions to local context;
7. The ‘right message’ is defined for the whole community. However, getting the message ‘right’ means tailoring for different stakeholders and audiences;
8. Messages and actions are solution oriented and designed to motivate positive changes and not to stigmatize any culture or behaviours;
9. Strategies and support services are designed to be sustainable and backed by policies and environmental changes;
10. Evaluation and monitoring are implemented at various levels. This is achieved through the collection of information on process, output and outcome indicators, and informs the future development of the programme.

### Organization

#### At central level

In each country, the EPODE programme has to be endorsed by central public authorities (ministries) and scientific organizations. This is critical to ensure that the programme is consistent with official recommendations and policies on diet, physical activity and health. To ensure the overall management of the programme at central level, EPODE is coordinated in each country or state by the CCT which brings together skills related to public health issues, from social marketing to network organization, professional training, general public communication and press relations. The CCT is also in charge of advocacy for the programme at central level and coordination of the evaluation and monitoring scheme (see Box 1).

**Box 1 Summary of the role of the EPODE Central Coordination Team**

- Advocacy for commitment and resource mobilization across federal/national/state/regional stakeholders: experts, ministries, health professionals, sports groups, scientific groups, non-governmental organizations, economic players (private companies, producers);
- Contact with candidate communities and agreement with local political leaders;
- Initial and ongoing training, coaching and experience-sharing sessions for the local project managers;
- Development of tools and materials based on social marketing to define themed messages, actions and stimuli:
  - Methodological tools for the local project managers;
  - Mobilization tools to be transmitted to the local stakeholders;
  - Communication tools to be disseminated at local level;
- External communication about EPODE through a communications plan which includes newsletters, a web site, media commentary, presentations and publications;
- Coordination of the programme evaluation and monitoring;
- Coordination of the scientific team activities and evidence for social marketing themes;
- Coordination of the leadership support activities such as the Mayor's Club.

The CCT operates with the advice of a multidisciplinary committee of independent experts in the fields of paediatrics, nutrition, psychology, physical activity, marketing, sociology, health communications and educational methods. Its role is to provide essential scientific support to the programme especially in defining priority topics for action, monitoring and evaluation.

In order to ensure the sustainable mobilization of human and technical resources at central level, the CCT is responsible for a long-range budgetary plan. The distribution of expenses at central level is related to the diversity of activities. This is also managed by the CCT.

**Figure 2:**
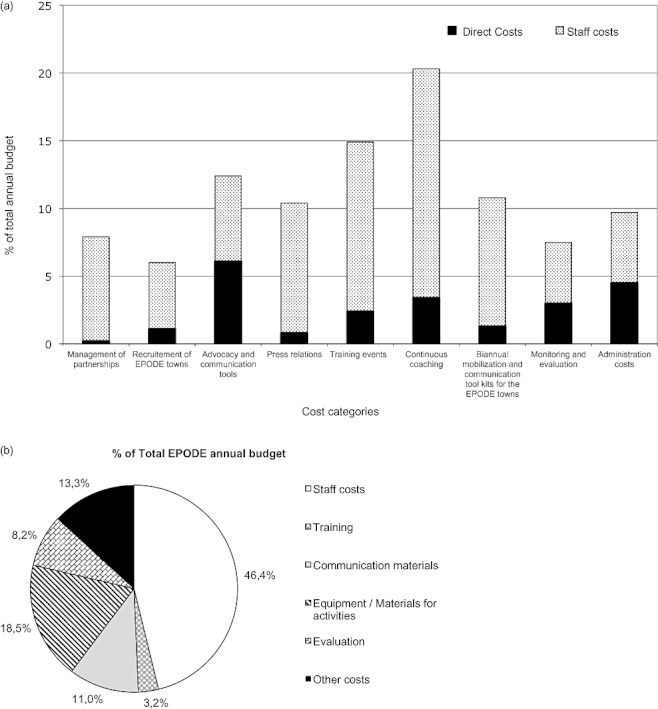
(a) Distribution of Ensemble Prévenons l'Obésité Des Enfants (EPODE) national expenses (France, 2009). This figure represents the distribution of the annual budget of the EPODE national coordination activities in 2009, i.e. €981,667. The programme concerned 226 communities counting 4,000,000 inhabitants. (b) Example of EPODE expenses distribution in a French pilot community in 2009. This figure represents the distribution of an annual budget of €95,000 in an EPODE town counting 50,000 inhabitants and including more than 3,000 children aged 5–12 whose body mass index is monitored in the EPODE framework.

Using the example of the EPODE programme in France, as detailed in Fig. 2a (226 communities involved to date), principal costs are usually related to staff (six people being employed full time). Direct costs are dedicated to the development of materials (web site, newsletters, communication and mobilization materials). Resources may come from public, private or mixed funds. Financial resources should ideally be provided from the public sector so that no commercial conflicts of interest arise. However, if insufficient public funding is available, the setting up of the programme should not be postponed if appropriate private funds can be mobilized. In some countries, private grants are of longer term (5 years at least) than public grants, which is an advantage for the sustainability of the programme. Governance rules should be made clear to preserve the public health goal and prevent possible conflicts of interest. If a private partner is involved, the relationship is governed by a long-term commitment charter, which guarantees mutual respect and trust for each party.

Private partners need to commit to (i) not intervening in the programme's content; (ii) not associating the EPODE programme with any promotion of a product brand; (iii) for the purposes of communication, only referring to the relationship as part of a corporate social responsibility commitment (the CCT organizes regular meetings with all funders involved at central level); and (iv) not displaying a logo of partners on any EPODE materials distributed in schools and other local childhood settings (as set out in recent recommendations by the World Health Organization and the International Obesity Task Force set up by the International Association for the Study of Obesity) (21,22).

#### At local level

Local administrations (education, health, sports, culture, etc.) will be more likely to become involved in the EPODE programme if it is perceived as a way to reach objectives set by the national public health strategy (Bergeron *et al*., unpublished). The commitment to EPODE must result from strong local political willingness to support and launch the project. Each community that is committed to the EPODE programme signs a long-term commitment charter (see Box 2).

**Box 2 Main community commitments to the EPODE programme**

Each community committed to the EPODE programme signs a charter in which it agrees:

- To be involved in the programme for at least 4 years;
- To appoint a councillor as a representative of the town council;
- To appoint a local project manager who will be the primary contact for the Central Coordination Team and will have the assignment of coordinating the programme at local level;
- To comply with the programme's philosophy and basic principles;
- To ensure that a dietician and physical activity teacher (or an activity leader with physical activity skills) are involved at various stages of the programme;
- To print and distribute the information and communication documents supplied by the Central Coordination Team to the target groups concerned (action sheets, general public information leaflets, posters, etc.);
- To build on the EPODE brand: having all new local initiatives approved by the Central Coordination Team
- To comply with and implement possible adaptation requests made by the Central Coordination Team concerning label requests.

Besides the municipal health services, many other departments, such as communication, educational and community life, sports, social affairs and community planning, are encouraged to become involved. EPODE offers the opportunity to develop a cross-municipal strategy, for each department to play a part, and to adapt certain professional practices and the local facilities and services delivering public health goals.

Local authorities ensure a significant and sustainable funding contribution for the local organization (see Fig. 2b), especially through the appointment of an EPODE local project manager, working in the field with a cross-cutting remit intended to foster local group dynamics (see Box 3). The local project manager takes responsibility for managing the multidisciplinary local steering committee of professionals in order to foster positive peer-to-peer dynamics and accelerate the implementation of local actions by a wide variety of local stakeholders (see Supporting Information Appendix S1).

**Box 3 The role of the EPODE local project manager**

- Be the link between the Central Coordination Team, its elected representatives and local stakeholders;
- Get the steering committee members on board and involved;
- Identify local stakeholders that are deeply involved with the local population and that can serve as ‘ambassadors’ for the EPODE programme;
- Strengthen existing initiatives as part of a network and initiate new actions;
- Advocate for the programme with the support of the municipal communication department and elected representatives;
- Get in contact with public and private partners that may fund or contribute to the project's funding by providing equipment, human resources, techniques, etc.;
- Coordinate the local monitoring (including the measurements of children's height and weight);
- Attend meetings that make possible the sharing of best practices with local project managers of other communities

The local project manager benefits from the political support of an elected representative of the community council who is a local decision-maker and spokesperson for the programme. This leader takes charge of facilitating the involvement of all municipal departments, the establishment of local partnerships and the implementation of actions. EPODE communities may also benefit from financial or ‘in-kind’ resources from private partners to implement specific projects or activities. They may also benefit from public grants awarded at the federal, national, state or regional level for the implementation of local activities. Private contributions to the budget vary according to the particular community, and this presents challenges in indicating an average budget.

An economic evaluation, which takes into account cross-cutting human resources mobilized for the project at local level (e.g. time invested by health professionals or teachers), is complex, because the philosophy of the EPODE methodology is to involve local stakeholders on a voluntary basis as part of their daily professional activities.

### Implementation

**Figure 3:**
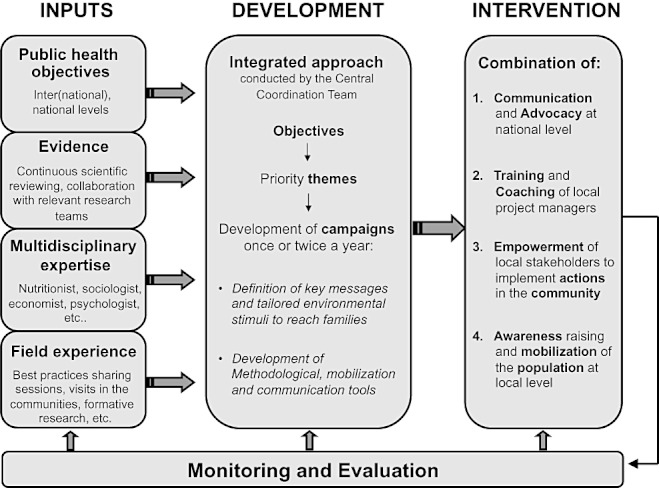
Ensemble Prévenons l'Obésité Des Enfants social marketing approach inspired from various existing frameworks (25,26).

In recent years, social marketing techniques have become widespread in community-based obesity prevention programmes to promote healthier behaviours. Previous reviews have shown that interventions using these techniques can achieve positive changes in risk behaviours (23,24). Individual cognitive-based strategies alone are unlikely to create sustainable behaviour changes, thus the aim of EPODE is to generate change to the social and physical environment, as well as individual education and personal change. From various existing frameworks (25,26), the social marketing approach of EPODE can be presented through different steps (see Fig. 3).

#### Structured input process

The CCT receives inputs from multiple sources, such as official recommendations, expert consultation and literature reviews. Field experience from EPODE teams also contributes. From the objectives and priority themes identified, campaigns of 6–9 months are planned. Each campaign highlights one specific theme at a time related to food and/or physical activity habits. The inputs identify barriers and levers for behaviour change among different target groups, as well as the evidence support for, and feasibility of, actions to be implemented.

#### Raising awareness

This process leads to the development of a mix of techniques and tools aimed at advocacy for the programme at central and local levels (see Table 1). For each EPODE campaign developed once or twice a year, communication tools are developed at central level in collaboration with local stakeholders and the expert committee. These tools are printed and disseminated in the communities, so that they directly reach the children and families. They focus on simple and solution-orientated key messages, and motivating positive behaviour changes without stigmatization. These tools have exciting execution elements, and colourful visuals, using cartoon characters adapted to appeal to children. They also include practical information adapted for parents (27). At the national level, a press office maintains contact with journalists and organizes interviews and broadcasts. This ensures a substantial amount of press coverage of EPODE actions at central level and in the communities. (In 2009 there were 450 press clippings relating to the French EPODE programme.)

**Table 1:** Overview of main EPODE tools developed at central level

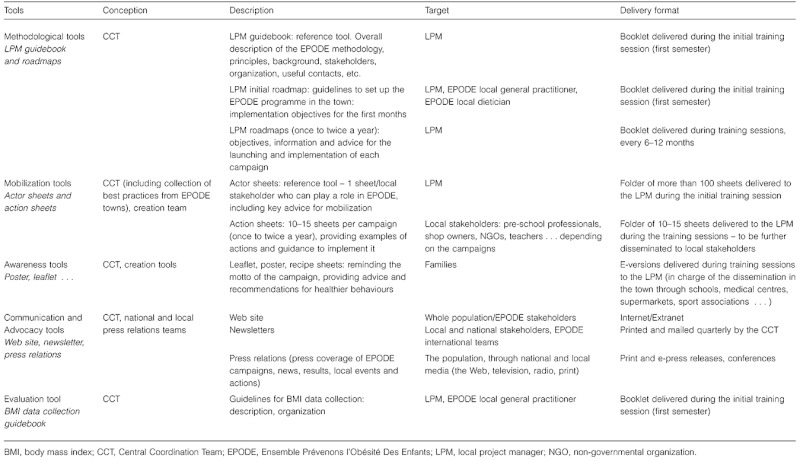

#### Empowerment of the local project manager and local stakeholders

The CCT visits each EPODE site at least once a year for specific follow-up and coaching and for local communication purposes. Regular training sessions for the local project manager are also organized at a central level three times a year, based on the themed campaigns. Capacity-building documents (see Table 1) are delivered to the local project manager during these sessions and help to foster local dynamics (e.g. partnerships and networks), empower local stakeholders to initiate local projects and activities (roadmaps, adaptable action sheets), develop local communication and press relations, and collect information on evaluation indicators (e.g. body mass index [BMI] measurements). The local project managers also have continuous contact with the CCT for advice on projects and actions to be initiated with local stakeholders (see Box 4). An annual EPODE congress encourages shared experiences and networking.

**Box 4 Examples of actions implemented by local stakeholders. The case of the campaign ‘Let's meet with the fruit’ (France, 2009–2010)**

Actions may be designed and organized during the campaign. These can take the form of drama lessons and artistic contests in leisure centres, fruit tasting in market places, walking trails with fruit tasting stops with local producers, afternoon fruit snack distribution in schools, fruit taste education workshops in pre-schools, fruit consumption favoured during a town's events (e.g. sports meetings and receptions) and distribution of materials to families (e.g. storybooks, recipes, magnets, etc.). Actions can similarly be built continuously or by recurring events that provide the opportunity to promote fruit consumption (e.g. tasting and cooking workshops with families in social centres, breakfast events in schools, open days of local producers and farms, annual EPODE week of local associations for nutrition and physical activity, etc.).

#### Changing environments

Local actions to change the local environments are also maintained throughout the year, although these may not be directly related to the ongoing EPODE campaign. These changes are planned through activities that are often pilot tested before being transferred and adapted to other communities and organized in a sustainable manner. The process is facilitated through the development of EPODE action sheets that provide practical information for the replication of the projects, such as information on budget, human and technical resources, process and timing, etc. (see Table 1). Examples of these include the optimization of the school catering service, as well as changes to physical environments, such as the rearrangement of school playgrounds, the installation of multisport courts in neighbourhoods, the development of baby gym facilities and activities, and improvements to the ‘walkability’ of the town.

### Creating the Ensemble Prévenons l'Obésité Des Enfants brand

To bring visibility and a sense of ownership, it is critical to create a singular brand whose features match the EPODE philosophy. This entails establishing central components to make EPODE stakeholders feel part of a common positive initiative, and to foster group dynamics and long-term commitment. For local stakeholders, it can be a lever to legitimize their action and involvement, and highlight a portfolio of initiatives under the same umbrella brand. The branding of EPODE materials also facilitates recognition of the programme by key ‘ambassadors’ as well as the general population, and creates a strong visual consistency across all communities.

### Monitoring and evaluation

EPODE evaluation and monitoring is implemented at various levels through the collection of information on process indicators (e.g. central partnerships, local steering committee meetings), output indicators (e.g. the number of local actions, participation of families and children) and outcome indicators (e.g. changes in dietary habits, childhood obesity prevalence) (see Table 2).

**Table 2:** EPODE main monitoring and evaluation levels, indicators and tools

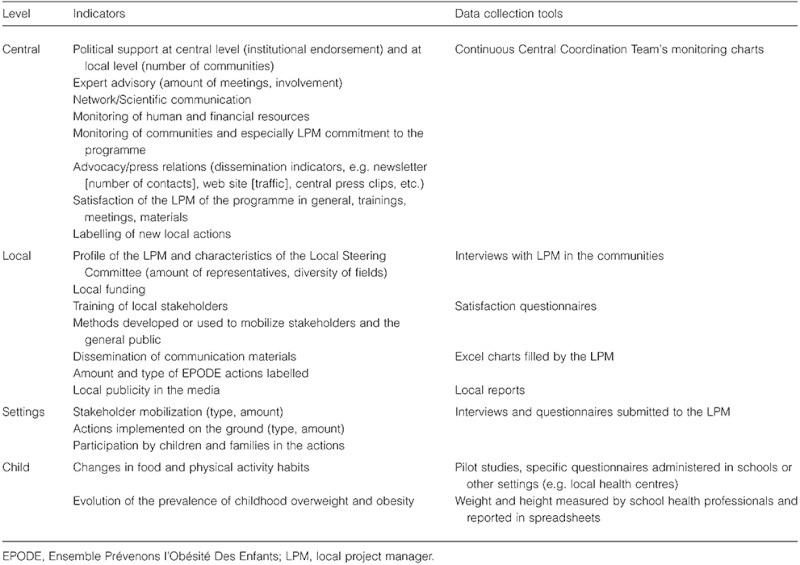

#### Anthropometric measurements

The monitoring and evaluation approach has so far included the collection of data on weight and height of all children (28). BMI data allow international comparisons (29) which is not the case for other adiposity markers in children (e.g. waist circumference or skin-folds). BMI data collection in children is also a key driver not only for politicians (as obesity prevalence is the principal goal for the EPODE methodology), but also for the mobilization of stakeholders at central and local levels. These data highlight the efforts and activities carried out and help to ensure a good media coverage of the programme.

#### Process and output indicators

The EPODE monitoring and evaluation approach complies with the recommendations by Doak *et al*. (14) who investigated school-based obesity programmes worldwide. An enhanced framework – including input, activities and output indicators – is now being established, based on learning from the academic literature and the experience within EPODE communities. ‘Input’ reflects all inputs required to support the programme, such as funding, partnerships, network, policy, knowledge and materials. ‘Activities’ reflect all action steps needed to produce the programme outputs, such as information, community development, advocacy and intersectoral action. ‘Output’ reflects everything directed at consumers or the target population, such as communication materials, courses and services.

#### Dissemination of results

Main results of evaluations are reviewed at the central level by the expert committee (depending on the set programme goals and programme objectives), and are then communicated to the local project manager or mayor of each community. They, in turn, are encouraged to communicate the outcomes at local level to both the general public and the stakeholders. Results are also communicated to partners at the central level and further disseminated through presentations at congresses and conferences, publications and to the media. The central coordination also provides assistance in linking media to designated EPODE sites when necessary.

## Ensemble Prévenons l'Obésité Des Enfants progress and international development

From a process designed by an EPODE international coordination team, EPODE programmes were launched in 2006 in Spain and in Belgium (THAO and VIASANO programmes, respectively). Similarly in 2008 a programme was established in Greece (PAIDEIATROFI programme), and more recently in South Australia (OPAL programme) and Mexico (EPODE-5 PASOS programme) (see Table 3).

**Table 3:** EPODE programmes in the world (2010)

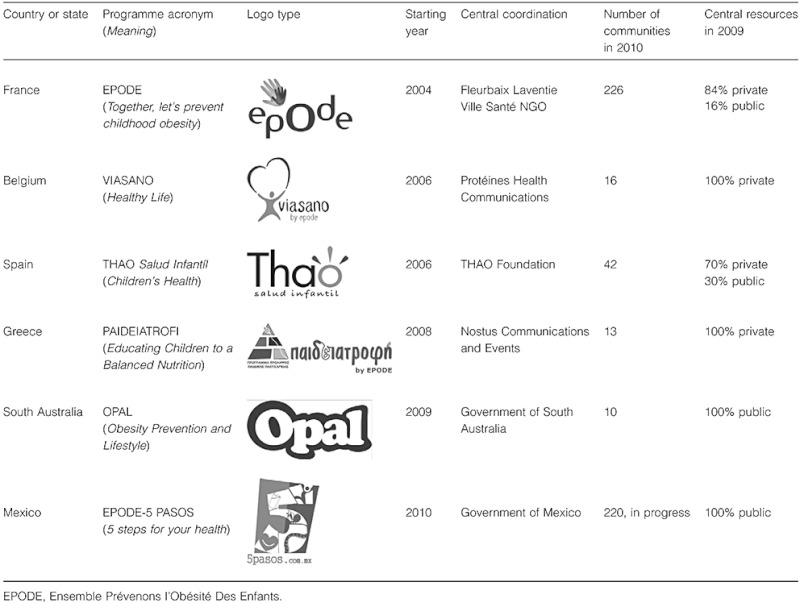

**Figure 4:**
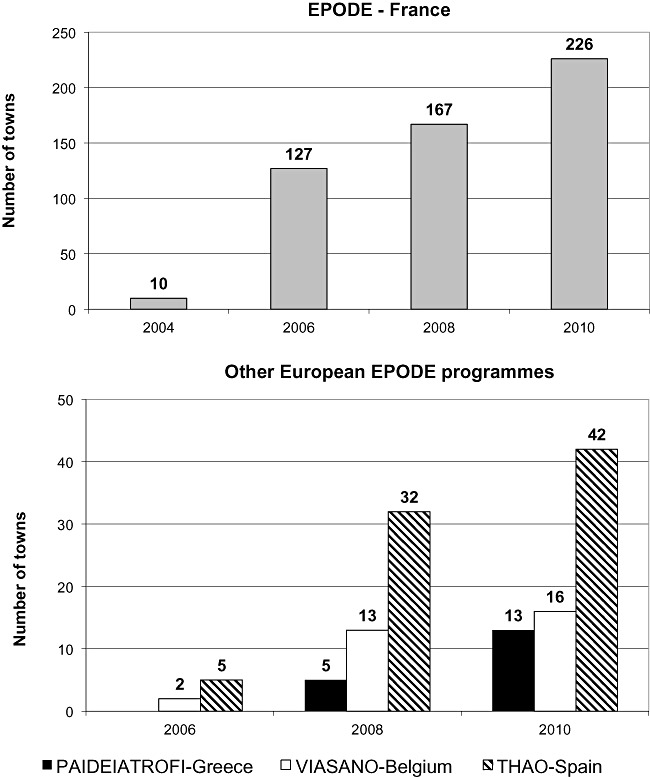
Evolution of the number of communities involved in Ensemble Prévenons l'Obésité Des Enfants (EPODE) France and other European EPODE programmes from 2004 to 2010.

More than 500 communities across the world are now part of the programme, and in France, 5 years after its launch, 90% of the original EPODE pilot communities are still active (see Fig. 4). Initiatives in other countries, such as the JOGG programme in the Netherlands (30) or the Healthy Weight Communities in Scotland (31), have been inspired and influenced by EPODE methodology.

The purpose has been to promote the core EPODE methodology in new countries or states, taking into account the particular political, cultural, scientific and administrative specificities, while ensuring that the approach is in accordance with EPODE principles.

### Resource mobilization

The EPODE mobilization of resources differs according to each country. This is due to the central coordinating entity determining the way resources are mobilized at both central and local levels, and the percentage of public and private funds that are channelled to the programme. EPODE resources at central level mainly come from either the support of corporate partners (as for VIASANO and PAIDEIATROFI), government sources (as for OPAL and 5 PASOS) or both (as for EPODE France, THAO) (see Table 3).

The annual budget at central level differs from one country to another, although in each case resources are planned to ensure coordination for 3–7 years, depending on the country. It is noteworthy, however, that the budget of the coordination team in France increased only slightly over 5 years, while the population size covered by the programme increased significantly: Estimations for 2004: a budget of €450,000 for 500,000 inhabitants (i.e. approximately €1 year^−1^ inhabitant^−1^). Estimations for 2009: a budget of €1 million for 4,000,000 inhabitants (i.e. approximately €0.25 year^−1^ inhabitant^−1^).

### Social marketing campaigns

While some campaigns are replicated from one country to another (see Supporting Information Appendix S2), the methods and social marketing process must be adapted to recognize specific barriers and utilize appropriate levers and opportunities for behaviour changes in each country.

### Evaluation practices

To date, all EPODE programmes have initiated continuous monitoring and evaluation practices at central, local and child levels, as well as in certain settings such as schools (see Table 2 and Supporting Information Appendix S3). Developing consistent practices and collecting comparable data across communities and programmes remains an important challenge.

In EPODE communities in France, a long-term collaboration with the Ministry of National Education makes it possible to involve school health professionals in the collection of weight and height data. This contribution is critical when considering the time and human resources necessary for such data collection. In the THAO programme, local team members (especially health professionals, students or research teams) collect data. In PAIDEIATROFI communities where it proved difficult to perform measurements in schools, alternative settings such as sports clubs or health centres are used. In Belgium, BMI data for the entire population of children are collected, and although official authorizations have to be renewed regularly to have access to the data, with three different communities using different methods, the screening of overweight and obesity is thus made easier in VIASANO communities. This approach gives the evaluation team access to comparison samples, while in Spain, the THAO coordination teams recently integrated 19 control communities, and in Australia OPAL is using matched communities for comparison. Comparison with national surveys is also a possibility, as has been adopted in France.

## Discussion and future challenges

The EPODE methodology has been described in terms of its four pillars: (i) political commitment; (ii) resources; (iii) support services; and (iv) evidence. For each pillar, the discussion section below highlights the achievements of EPODE and its future developments.

### Political commitment

To have any chance of success, it is commonly observed that local authorities have to engage in the EPODE programme on a voluntary basis. As noted in a recent research study, the continuous and long-term implementation of activities in communities, together with the support of local political will, is critical for successful institutionalization of the EPODE programme at local level (Bergeron *et al*., unpublished).

To be involved in the programme, French communities often contact the EPODE CCT. An active policy of recruitment is often not necessary because the reputation of EPODE travels through peer-to-peer awareness (especially through elected representatives), official reports and the media. In addition, mayors of the first 10 French pilot towns have created an independent NGO called ‘Club des Maires EPODE’ (EPODE mayors' club), which meets twice a year to share experiences as well as to raise awareness of the EPODE methodology with – other mayors. This organization aims to develop awareness of the importance of political leadership within EPODE, to foster cross-sectoral mobilization of politicians and civil servants in each community, and to share examples of successes and failures. All the communities joining the programme in France are now invited to be part of this NGO.

Politicians can be strong advocates of the programme at regional/state or national/federal levels and can mobilize many partners, networks of associations and private actors. As EPODE mayors and members of parliament, they can also mobilize their peers for amendments related to the food and physical activity environments. Conversely, if the elected representatives are not heavily involved initially, there may be a lower recognition of the actions undertaken by the local stakeholders, and thereafter a lower interest in getting involved. It is important to involve elected representatives from different political parties to provide continuity in the event of political turnover.

Apart from political leadership, the appointment of a project manager in each community is a compulsory commitment. There is no standard community size to implement EPODE; however, if necessary the project manager can be assisted by subproject managers in some areas (e.g. in the city of Paris, a global project manager has been hired for an area of three districts and approximately 2,000,000 inhabitants, while each individual district has its own project manager).

This particular model has raised questions regarding the politicians' motivation for involvement. While in part the answer may be re-election, it is also apparent that they are responding to the expectations of their voters to find solutions to the recognized problem of obesity. In that sense, childhood obesity prevention can be seen as a deserving and common challenge across political borders, and engaging in EPODE could appear to be a relatively cheap and visible solution. For elected representatives, EPODE is a tangible way to improve health, tackle social inequalities and foster physical environment changes in the community.

It is noteworthy that while the time frame for action may be short for politicians (they need immediate visibility and results), it is longer for scientists (32) and this can become a point of tension. Another potential point of discord is the time frame for dissemination of results. Politicians frequently expect short-term communications, highlighting the success they may have in their own community without considering issues of the statistical significance of their local results. Nonetheless, measurements of obesity prevalence in the community may be a good opportunity to reinforce awareness of the childhood obesity issue, legitimize the action and encourage mobilization of resources.

### Resources

Public–private partnerships (PPPs) are one of the mechanisms through which healthy lifestyle promotion initiatives are addressing the childhood obesity problem worldwide (33,34). PPPs in EPODE can be seen as an ‘organisational cooperation project’ in which different skill sets are brought together.

For public partners and NGOs, PPPs contribute to the sustainability of EPODE and other similar programmes, especially from the funding provided. For private partners, PPPs are important for corporate social responsibility and public relations by building trust among consumers and political authorities. However, the additional value is not only in terms of money; at a local level, building action with local private partners (supermarkets, local companies, producers, retailers, etc.) can lead to a synergy of micro-actions and may provide financial means, specific skills or ‘in-kind’ resources (e.g. equipment). Communication regarding their support to the programme can only occur at local level, and cannot be related to the promotion of any brand or product. However, public parties sometimes see the private sector as threatening because it is perceived to be motivated mainly from a profit perspective, which is thought to conflict with health promotion activities. Furthermore, the public sector may be afraid of losing its integrity and independence when partnering with the private sector, which may cause conflicts of interest. The private sector may perceive the public sector as bureaucratic, politically dependent and slow moving, which may be more visible where programmes are entirely public funded. The private sector may also consider that its own commitment is not sufficiently highlighted. Another risk can arise if the private partners at national and local levels are different. This may create tension among private partners.

PPPs may also present different opportunities and risks to both parties. In the literature, both negative and positive considerations are being expressed and suggestions have been made to develop new kinds of PPPs in public health (35–39).

A committee of the EEN project, supported by the European Commission (DG Health and Consumers) from 2008 to 2011, is developing recommendations for the PPP management in EPODE (20,40).

### Support services

The determinants of obesity at a population level are complex (41). Conscious and unconscious behaviours related to diet and physical activity are interrelated across levels and sectors. Accordingly, intervention requires multilevel, multisectoral and multistakeholder preventive strategies (42).

Behavioural models have proven to be valuable frameworks for the design of successful health promotion programmes (43) and support the argument that the family and environment are crucial for the development of food and taste preferences, patterns of food intake, eating styles, activity preferences, etc., that shape children's weight status (44). The EPODE methodology aims to empower local stakeholders, giving them sufficient trust and flexibility to operate, at local level, tailoring their actions based on the profile and needs of the population. EPODE support services aim to create the necessary conditions in families for the sustainable dissemination of messages and implementation of change so that healthier behaviours can be adopted.

These services include a broad range of activities at central level (advocacy, partnership management, training and coaching of local project managers, development of social marketing campaigns, press relations, monitoring and evaluation, etc.) and at local level (political leadership, steering of projects, coordination of actions, data collection, communication, etc.). In particular, social marketing is an important component of the EPODE methodology. This is in contrast to public health programmes that have often been based on the capacity of individuals to give rational consideration to the recommendations they are given. Growing evidence indicates that interventions using social marketing principles can be effective and promising (23,25,45,46) and core communication principles should also be considered (27). Accordingly, the core principle is to make changes easier, popular and sometimes humorous. The motivation should be built on actual insight and direct benefit for the target audience, based upon issues that touch their emotional perceptions. Changes in the social environment through the creation of network dynamics by involving all possible stakeholders are another core element of the methodology, which has shown to be effective in many different settings (26,47).

While positive dynamics can be observed in EPODE communities as a result of these activities (e.g. long-term commitment of local authorities, satisfaction of local stakeholders, good participation rates of the population), the nature and characteristics of chain reactions leading to best impact in the field in terms of behaviour changes still needs to be more fully documented.

Research by Organization for Economic Co-operation and Development (48) indicated that ‘key drivers of success’ of preventive interventions include high participation (in supply and demand), long-term sustainability of effects, ability to generate social multiplier effects (e.g. through the involvement of stakeholders in ‘different forms of dialogue and partnerships’ and ‘effective channels of communication’) and a combination of multiple interventions.

In order to have a deeper insight into EPODE methods, the EEN has initiated descriptive research of EPODE central and local coordination mechanisms, underpinning positive outcomes and challenges (20,40).

### Evidence

Concrete, practical and effective implementation of EPODE methodology requires the use of multiple sources of evidence such as ongoing scientific reviews, consultation with experts, interviews with local project managers, pre-testing of messages, monitoring and evaluation of actions at all levels.

Monitoring the evolution of dietary and physical activity habits in children is carried out in an increasingly comprehensive way, but sample size, consistency, accuracy, reliability and availability of human resources to submit questionnaires remain challenges. Existing frameworks are available in order to learn and understand how cognitive and environmental determinants work and could act as potential moderators or mediators between underlying causes and energy-balance-related behaviours (49,50). If resources are available, additional research could use data from these questionnaires as a basis to tailor interventions (specific subgroups such as ethnic minorities or high-risk groups), to study determinants, and to compare changes in body composition and health behaviours within and between EPODE communities.

In order to be able to monitor EPODE as a standardized approach, the future aim for EPODE communities is to have as a minimum a standardized set of questions in an EPODE questionnaire on energy-balance-related behaviours and determinants.

A great deal is still to be learned from CBIs, and the broad and sophisticated EPODE methodology with its enormous reach and potential impact provides strong opportunities not only to collect data but also to monitor and evaluate the EPODE methodology within and between EPODE communities and programmes.

A future challenge to the EPODE monitoring and evaluation framework is to define which indicators all EPODE communities should include, together with a more flexible set of indicators that may vary across EPODE communities. The importance of the monitoring tools to be chosen and the value of items in the EPODE questionnaires should be shared by local project leaders and partners. This is due to monitoring requiring a certain level of intrinsic motivation that exists only when practitioners realize that their programmes will be more effective and reach more people if they are able to adjust and improve their programmes on the basis of available data.

The evaluation and dissemination framework needs to be flexible and dynamic, with consideration for the feasibility in the field and that includes core and optional components. Most importantly it should be used as a stimulus for local EPODE teams and their members to adapt and change the programming where needed and/or appropriate. Future research and evaluation needs to take into consideration whether other evaluation designs are similarly useful, or indeed, whether they are a better alternative to the classic randomized controlled trial design.

## Conclusion

EPODE is the largest global childhood obesity prevention programme. The theory behind EPODE methodology reflects the multifactorial approach important in the prevention of childhood obesity. The four pillars show the core components of the approach and help to explain why EPODE has been able to reach this scale. Early evaluation of the EPODE methodology suggests encouraging results (28). Further important lessons are to be learned from a detailed evaluation of EPODE. Childhood obesity prevention programmes, which aspire to have a wide reach, may benefit from the insights into the EPODE methodology. Already EPODE is referenced in several national and international reports, position papers and conferences as an innovative example of a community-based project aimed at promoting healthy behaviours in children (19,^51^–64). Further insights will follow from the standardization of actions and monitoring across all EPODE programmes around the world.

## Abbreviations

BMI: Body Mass Index
CBIs: Community-based Interventions
CCT: Central Coordination Team
EPODE: Ensemble Prévenons l'Obésité Des Enfants
EEN: EPODE European Network
FLVS: Fleurbaix Laventie Ville Santé
NGOs: Non-governmental Organizations
PPP: Public–Private Partnership

## Supporting Information

Additional Supporting Information may be found in the online version of this article: **Appendix S1.** Categories of local stakeholders invited to be represented in the EPODE local steering committee and professionals/organizations to get involved in the community. **Appendix S2.** EPODE campaigns developed since 2004 in France (40), Spain (THAO), Belgium (VIASANO), Greece (PAIDEIATROFI) and South Australia (OPAL). **Appendix S3.** Data collected at child level in European EPODE programmes (BMI, body mass index). Please note: Wiley-Blackwell is not responsible for the content or functionality of any supporting materials supplied by the authors. Any queries (other than missing material) should be directed to the corresponding author for the article.
