# Supplementary material for: EPODE approach for childhood obesity prevention: methods, progress and international development
Source: Obes Rev. 2012 Apr;13(4):299–315. doi: 10.1111/j.1467-789X.2011.00950.x (PMC3492853; doi:10.1111/j.1467-789X.2011.00950.x)
Supplement: Supplementary file 1 — Additional Supporting Information may be found in the online version of this article: Appendix S1. Categories of local stakeholders invited to be represented in the EPODE local steering committee and professionals/organizations to get involved in the community. Appendix S2. EPODE campaigns developed since 2004 in France (40), Spain (THAO), Belgium (VIASANO), Greece (PAIDEIATROFI) and South Australia (OPAL). Appendix S3. Data collected at child level in European EPODE programmes (BMI, body mass index). Please note: Wiley-Blackwell is not responsible for the content or functionality of any supporting materials supplied by the authors. Any queries (other than missing material) should be directed to the corresponding author for the article. [file obr0013-0299-SD1.doc]

**Appendix 1:** Categories of local stakeholders invited to be represented in the EPODE local steering committee and professionals/organisations to get involved in the community

| **CATEGORIES** | **PROFESSIONALS/ORGANISATIONS** |
| --- | --- |
| **Health Professionals** | **Town or Hospital Centre specialists**: Pharmacist, General practitioner, Specialist physicians, Independent/Municipal dietician, Independent nurse, Midwife, Psychomotor therapist, Physiotherapist, Psychologist. **Occupational General practitioner** (Inter-company organisations or Intra-company) |
| **Local companies, retailers and producers** | Chamber of Commerce and Industry/Agriculture, Retailer and Craftsmen Associations, “Food” retailers or hypermarket/supermarket stands, Local producers, Restaurants (traditional, fast-food), local companies for support and a partnership in the community |
| **Political decision makers** | **Community level**: Mayor, Elected representatives (Urban planning, Education and school environment, Health, Social and Solidarity activities, Infancy, Transportation, Youth, Sports and Leisure, Culture), Children’s/Youth’s Municipal Council. **Upper levels**: politicians at regional, state, national/federal levels |
| **Health organisations** | Health Insurance Funds (Regional and Local agencies), Local and Regional Directorates for Health, Social Affairs, Youth and Sports, Family Allowance Office, Local and Regional health education and promotion associations, Regional Health Observatory, Regional Public Health Group |
| **Pre-school** | Maternal and Infantile Protection Services, Children’s Social Aid Services, Day care centres, day nurseries, Childminders, pre-school sports associations (e.g. Baby gym clubs) |
| **Social Network** | Sports associations, Charities and food relief associations, Service clubs, Educational farms, Farmer’s assistance association |
| **Local actors involved in community’s facilities** | Community Centres, Hospitals, health care and prevention, sports facilities (Stadiums, pools, gymnasiums, etc.), Fitness trails and courses, Cultural facilities: Youth and Cultural Centre / Neighbourhood centre, Libraries and/or multimedia centres, Museums |
| **Media and communication** | Local and freelance journalists, Community’s communication services |
| **Extra-curricular organisations** | Leisure/recreation centres (Managers, Activity Leaders) |
| **School catering** | School catering manager and staff, **If self-managed**: Kitchen chef and staff, **If sub-contracted**: Service company, The company’s dietician |
| **Schools** | Chief Education Officer, school health professionals, Education counsellors, Sport youth workers, School teachers, local authority employees specialized in nursery schools, Parent-teacher associations |
| **Other local actors** | **Universities, graduate schools and their students** (e.g. Public health university, Sports university, Dietetics school, Arts School, Agricultural School, Hotel and Restaurant Management schools, Agronomics Research Institute, Nursing care schools. **Other local bodies** (e.g. Regional Directorate of Food, Agriculture and Forestry) |
