# Supplementary material for: EPODE approach for childhood obesity prevention: methods, progress and international development
Source: Obes Rev. 2012 Apr;13(4):299–315. doi: 10.1111/j.1467-789X.2011.00950.x (PMC3492853; doi:10.1111/j.1467-789X.2011.00950.x)
Supplement: Supplementary file 2 — Additional Supporting Information may be found in the online version of this article: Appendix S1. Categories of local stakeholders invited to be represented in the EPODE local steering committee and professionals/organizations to get involved in the community. Appendix S2. EPODE campaigns developed since 2004 in France (40), Spain (THAO), Belgium (VIASANO), Greece (PAIDEIATROFI) and South Australia (OPAL). Appendix S3. Data collected at child level in European EPODE programmes (BMI, body mass index). Please note: Wiley-Blackwell is not responsible for the content or functionality of any supporting materials supplied by the authors. Any queries (other than missing material) should be directed to the corresponding author for the article. [file obr0013-0299-SD2.doc]

**Appendix 2:** EPODE campaigns developed since 2004, in France , Spain (THAO), Belgium (VIASANO), Greece (PAIDEIATROFI) and South Australia (OPAL)

| **Years** | **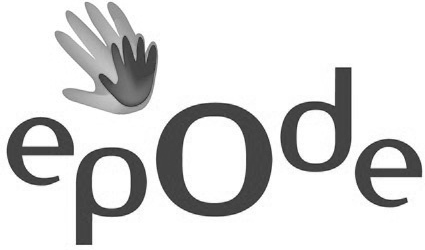** | **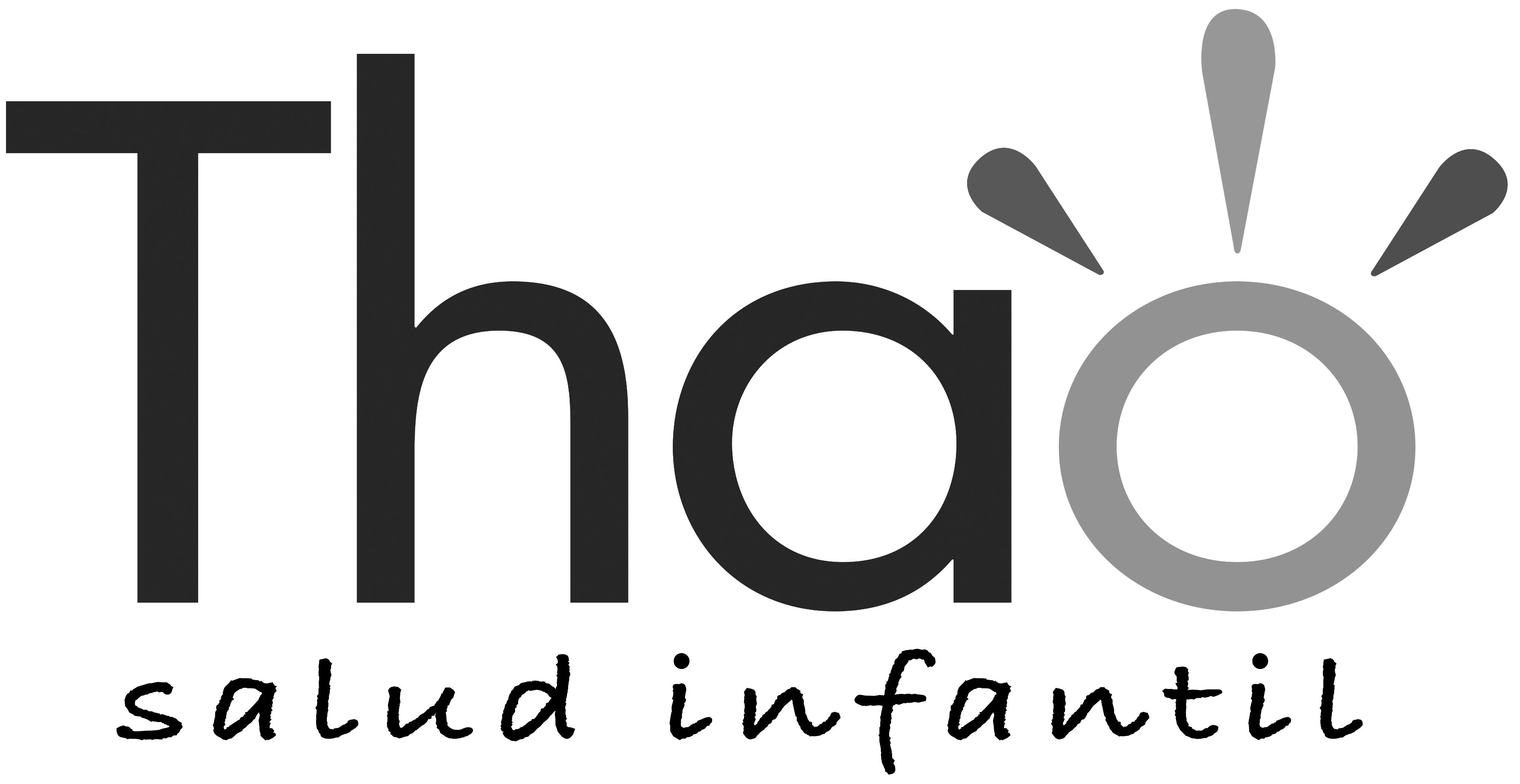** | **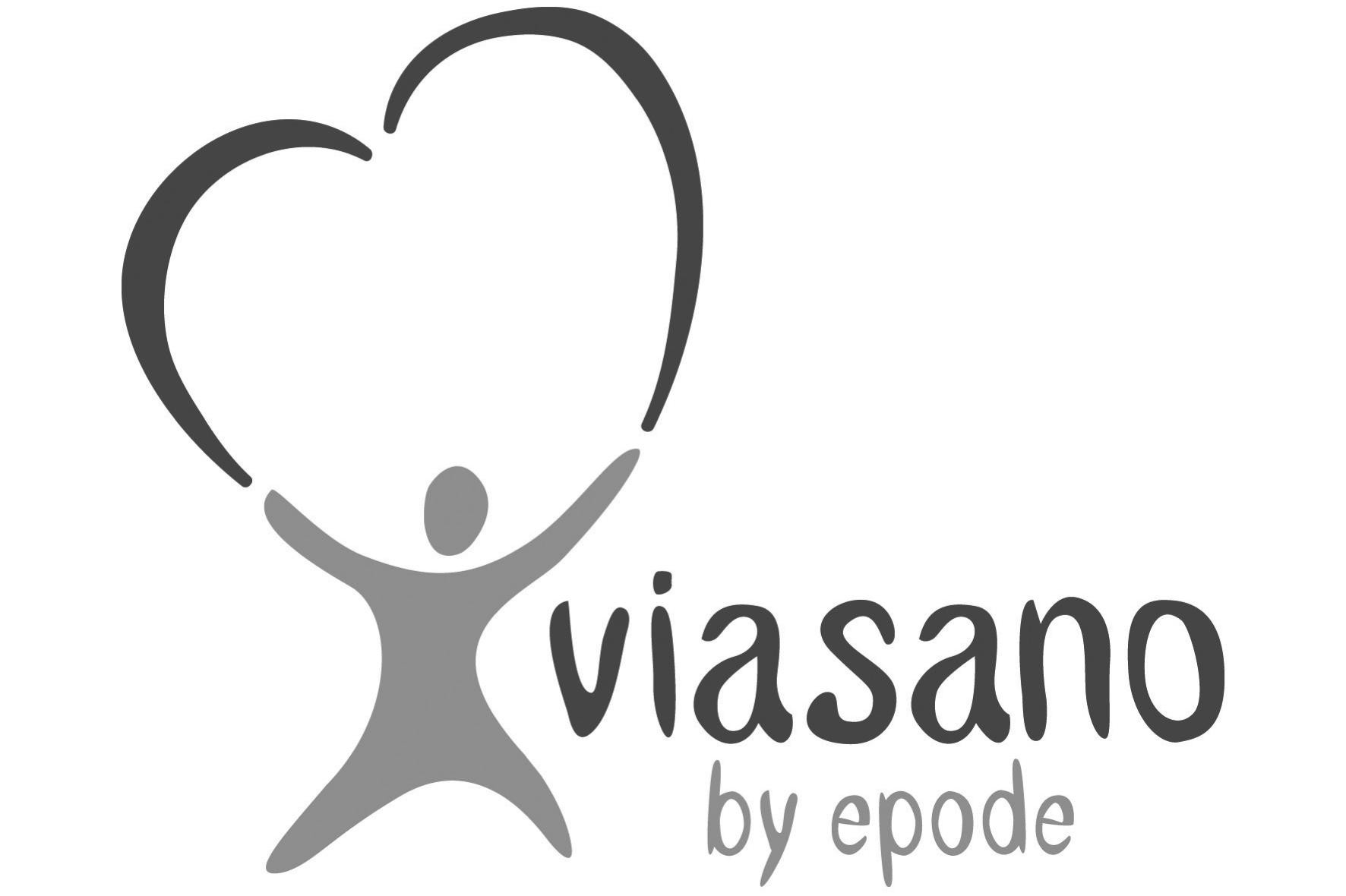** | **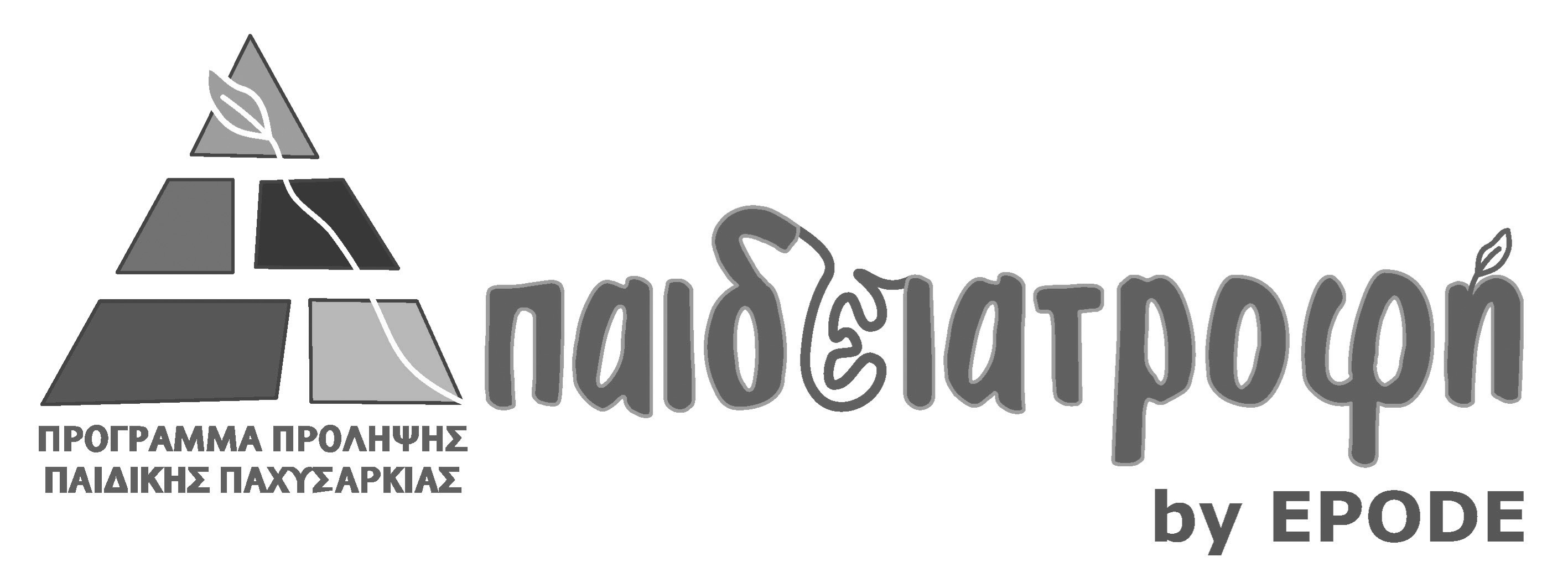** | **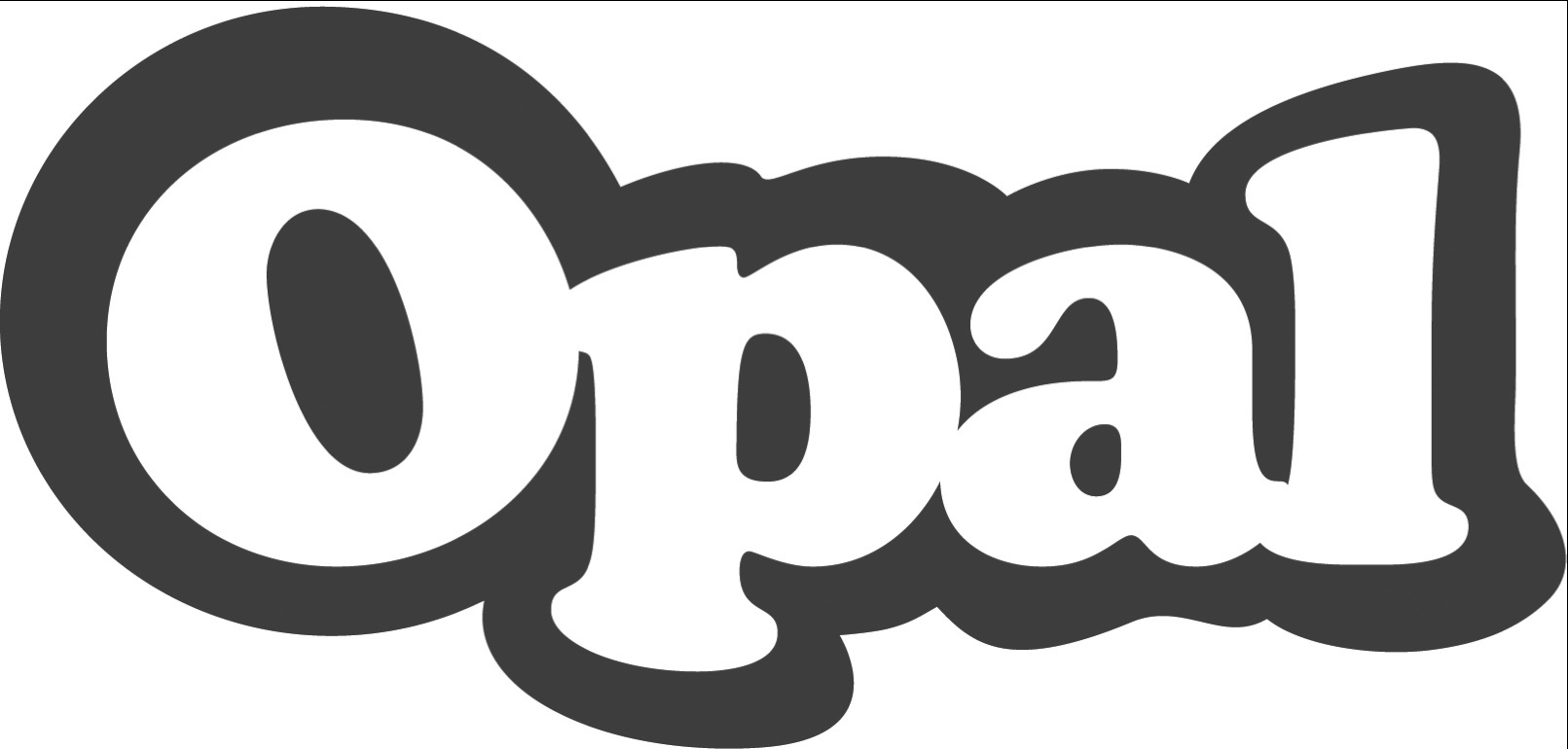** |
| --- | --- | --- | --- | --- | --- |
| **2004** | - The season has the taste for **vegetables** |  |  |  |  |
| **2005** | - The season has the taste for **starchy foods** - The season has the taste for **dairy products** - The season has the taste for **fruit** |  |  |  |  |
| **2006** | - Let’s enjoy **treats** with moderation - We **move** and we like it! - **Easy**, **cheap** and **healthy** eating? It's to be cooked! |  |  |  |  |
| **2007** | - Let’s enjoy **water**! - Health starts **at the table** | - The season has the taste for **fruit** | - **Fruit** are good for us! - Are you **active** or **sedentary?** |  |  |
| **2008** | - **Playing** is already moving! - **Fish** has everything good | - Let’s learn about **water** and other **drinks** - Let’s learn about **starchy foods** | - **Dairy** products, for pleasant changes! - Be **active** as a **family** - **Vegetables,** let’s go for **more!** - **Move** at **school**, at **work** |  |  |
| **2009** | - **Physical activity**, everyone gets into it! - Let’s meet with the **fruit!** | - **Fruit** and **Vegetables**: 5 per day… Set the pace! - **Dairy products**, 2 or 3 per day and…**let’s move**! | - How to manage **sweets** and **crisps**? - Be **active**, whatever the weather is! - **Meals** - Fewer **screens**, more **movements** | - **Movement** and Physical Exercise on a daily basis - The season has the taste for **fruit** |  |
| **2010** | - Small, Medium, Large: At mealtime, adapted **portion sizes** to each one | - 3,2,1…Let’s go! **Physical activity** is fun and healthy! - Blub, Blub, Blub… Let’s dive into a healthy sea! : **Fish** and **seafood** season | - Light-handed **fat consumption!** - **Sleep** / slumber | - **Breakfast**, my secret weapon! - The season has the taste for **vegetables** | - **Water**, the original cool drink! - Give the **screen** a rest, **Active play** is best |
