# Supplementary material for: EPODE approach for childhood obesity prevention: methods, progress and international development
Source: Obes Rev. 2012 Apr;13(4):299–315. doi: 10.1111/j.1467-789X.2011.00950.x (PMC3492853; doi:10.1111/j.1467-789X.2011.00950.x)
Supplement: Supplementary file 3 — Additional Supporting Information may be found in the online version of this article: Appendix S1. Categories of local stakeholders invited to be represented in the EPODE local steering committee and professionals/organizations to get involved in the community. Appendix S2. EPODE campaigns developed since 2004 in France (40), Spain (THAO), Belgium (VIASANO), Greece (PAIDEIATROFI) and South Australia (OPAL). Appendix S3. Data collected at child level in European EPODE programmes (BMI, body mass index). Please note: Wiley-Blackwell is not responsible for the content or functionality of any supporting materials supplied by the authors. Any queries (other than missing material) should be directed to the corresponding author for the article. [file obr0013-0299-SD3.doc]

**Appendix 3:** Data collected at child level in European EPODE programmes *(BMI=Body Mass Index)*

| **Programmes** | **Data collected/ Indicators** | **Frequency** | **Sample** |
| --- | --- | --- | --- |
| **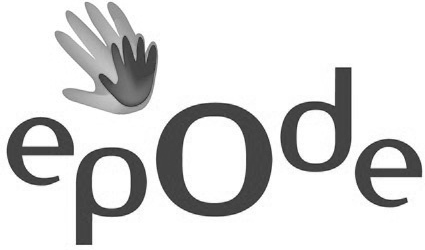** | - Evolution of children Body Mass Index (BMI) (aged 4–5 to 10–11) | Yearly for pilot communities,  Every 2 years for others | 50817 children  (27954 from 2005 to 2009, pilot communities)  22863 from 2007 to 2008, other communities) |
|  | - Evolution of the total subscriptions to physical and sports activities in the town - Changes in food and physical activity habits | During pilot projects or studies (2009-2010) | Samples of 30 to 500 children participating in specific activities |
| **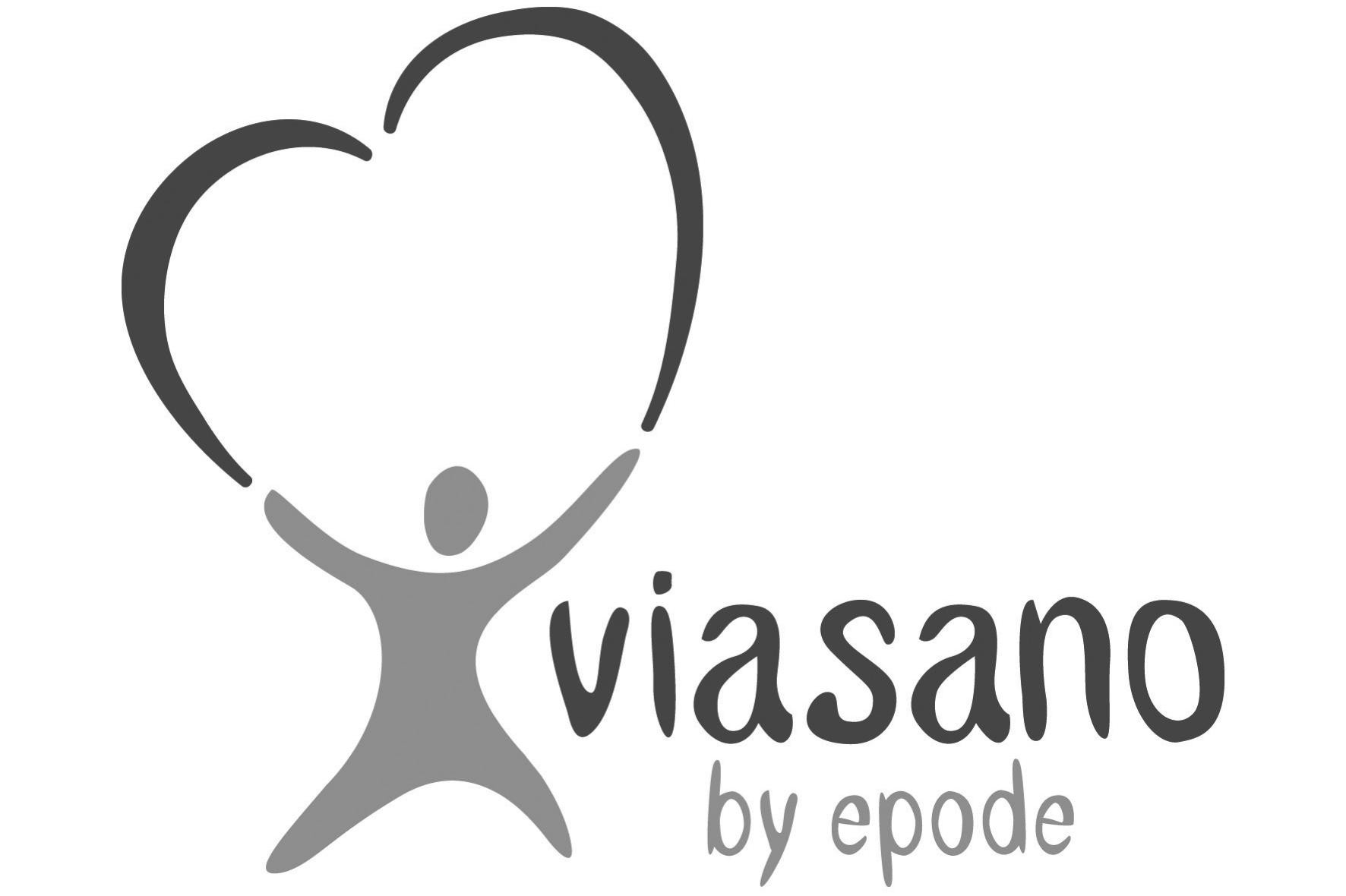** | - Evolution of children BMI (aged 4–12) | Every 2 years | 5,601 children in Flanders (2008), 1,920 children in Wallonia (2008), 2,810 children in the German-Speaking Community |
| **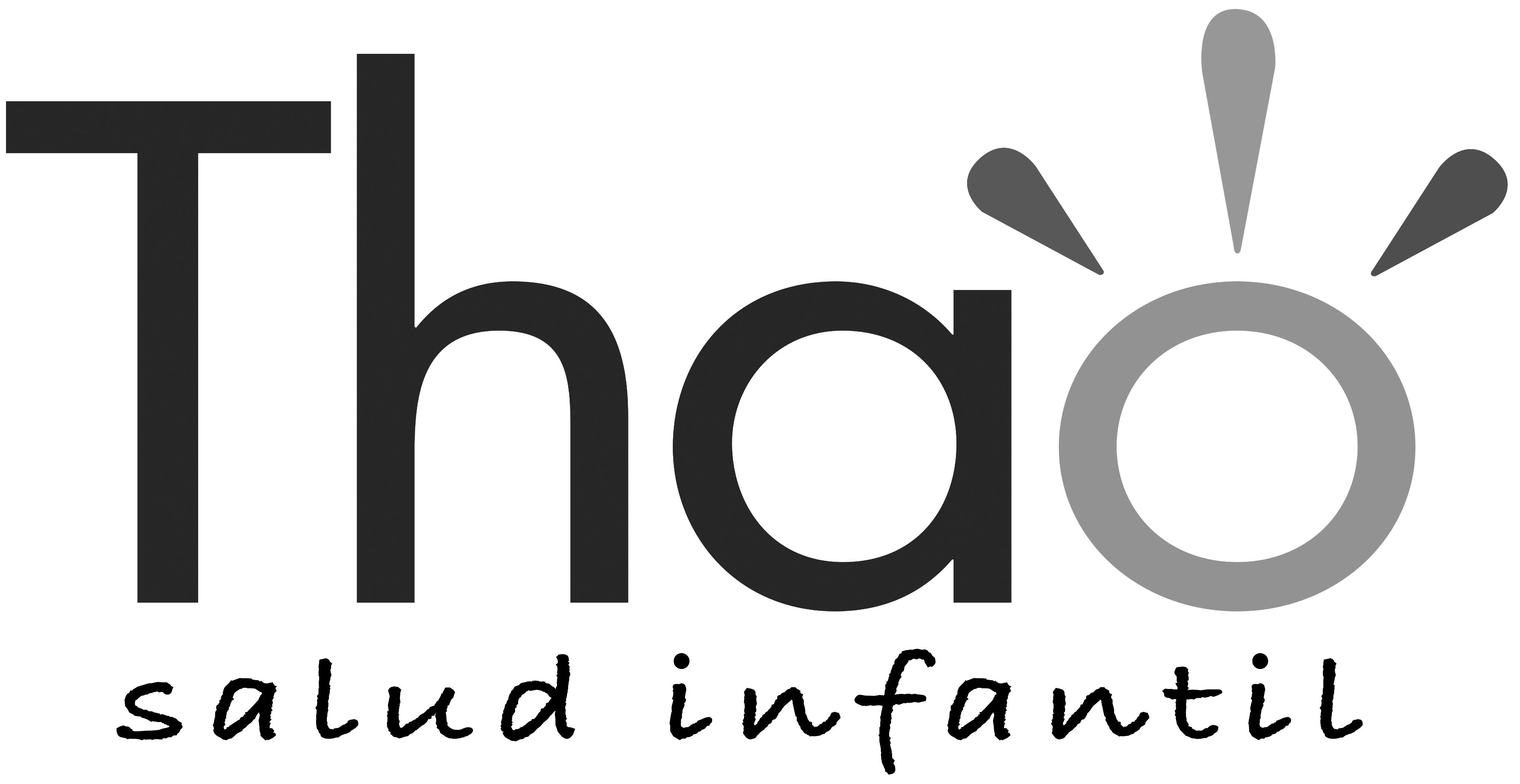** | - Evolution of Children BMI and Waist Circumference (aged 3–12) | Yearly | BMI: 8,880 children in 2007–2008 and 1,7088 in 2008–2009 / WC: 6,632 in 2008–2009 |
|  | - Diet and Physical habits (aged 8–12) | Yearly | 739 from 6 different communities and regions |
| **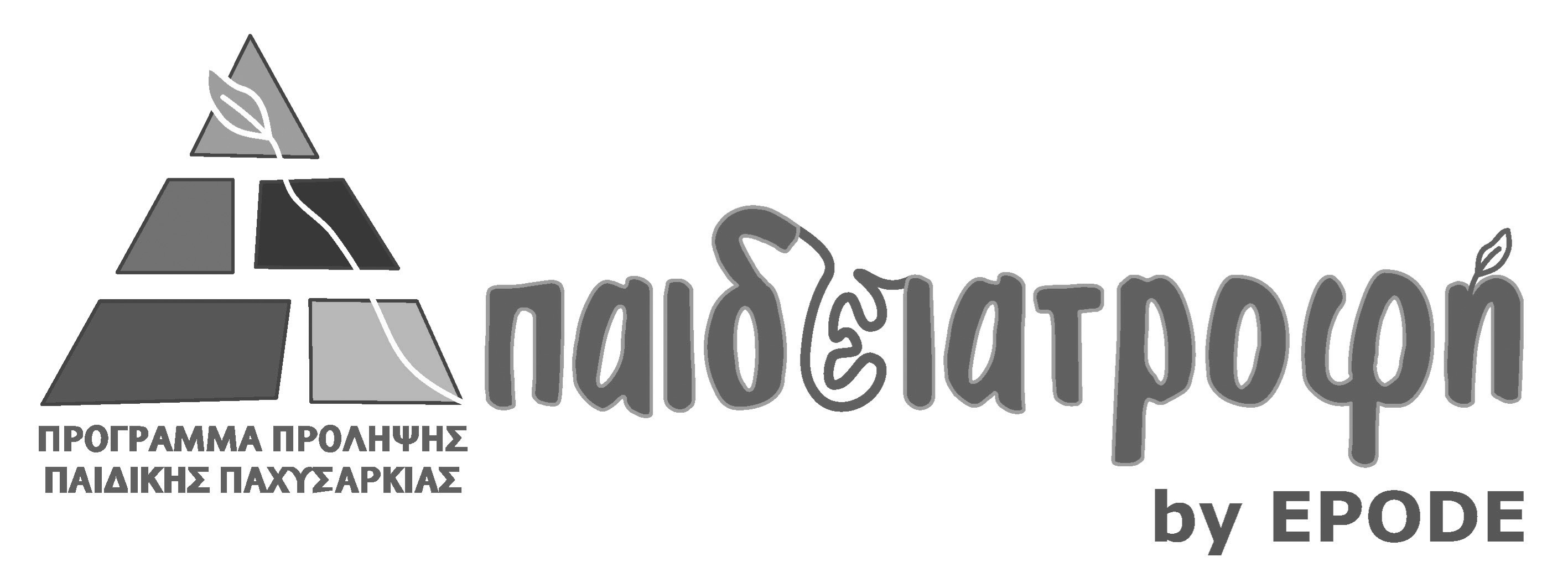** | - Evolution of children BMI | Start and end of programme | In progress |
